# The effect of Schroth Therapy combined with spinal manipulation for the treatment of adolescent idiopathic scoliosis

**DOI:** 10.12669/pjms.41.2.11355

**Published:** 2025-02

**Authors:** He Chen, Chaonan Zhang

**Affiliations:** 1 He Chen Department of Orthopaedics, Wenzhou TCM Hospital of Zhejiang Chinese Medical University, Wenzhou, Zhejiang Province 4325000, P.R. China; 2 Chaonan Zhang Department of Acupuncture and Moxibustion, Wenzhou TCM Hospital of Zhejiang Chinese Medical University, Wenzhou, Zhejiang Province 4325000, P.R. China

**Keywords:** Adolescent idiopathic scoliosis, Cobb angle, Schroth method, Spinal manipulation

## Abstract

**Objective:**

To evaluate the effectiveness of the Schroth method combined with spinal manipulation treatment in patients with adolescent idiopathic scoliosis (AIS).

**Methods:**

This was a single-center retrospective study performed between January 2023 and February 2024, in which 150 patients with AIS were treated with Schroth method with or without spinal manipulation at Wenzhou Traditional Chinese Medicine Hospital. Patients were classified into a study group and a control group with 50 patients in each group after screening. Intervention effects, maximum Cobb angle, clavicle angle before and after the treatment, trunk rotation angle, vertebral rotation angle, and lumbar range of motion (range of motion for lumbar extension and flexion) were compared between the two groups.

**Results:**

The overall efficacy of intervention in the study group was higher than that in the control group (96.00% *versus* 84.00%) (*P*<0.05). After the treatment, the maximum Cobb angle, clavicle angle, angle of trunk rotation (ATR), and vertebral rotation angle of the two groups decreased compared to pretreatment levels and were significantly smaller in the study group compared to the control group (*P*<0.05). After the treatment, the degree of lumbar extension and flexion in both groups increased compared to before treatment and was markedly greater in the study group (*P*<0.05).

**Conclusions:**

In patients with AIS, combining the Schroth method and spinal manipulation treatment was more effective in reducing the maximum Cobb and clavicle angles, trunk rotation angle, and vertebral rotation angle and restoring the lumbar range of motion compared to the Schroth method alone.

## INTRODUCTION

Adolescent idiopathic scoliosis (AIS), the most common form of scoliosis, accounts for approximately 80%-85% of all scoliosis cases.^1^ AIS affects approximately 2%-4% of adolescents between ages 10 to 18.^2^,^3^ In recent years, the incidence of AIS has been on the rise and the number of patients with low back pain has increased dramatically.^4^,^5^ AIS negatively impacts the physical and mental health of patients and, in severe cases, may also affect organ function and development.^4^–^6^

The treatment methods available for AIS include surgical correction and non-operative options, such as the Schroth method or the manual spinal manipulation intervention. The Schroth method, one of the most popular physiotherapeutic scoliosis-specific interventions in AIS, can effectively correct physical coordination, enhance spinal motor control and sensation, and prevent abnormal exacerbation of scoliosis curves in patients with AIS.^7^ Manual spinal manipulation techniques, which are also commonly used in the treatment of scoliosis, were shown to improve the function of the musculoskeletal system by adjusting the structural disorder of the spine.^8^ Studies show that this method can alleviate pain, correct and improve scoliosis, restore lumbar range of motion, and thus inhibit the progression of the disease.^8^,^9^

However, there is currently limited literature on the impact of the combined application of the Schroth method and spinal manipulation intervention on the functional rehabilitation of patients with AIS. This retrospective study aimed to evaluate the efficacy of the combined intervention and provide updated references for the clinical treatment of patients with AIS.

## METHODS

This was a single-center retrospective study. Between January 2023 and February 2024, 150 patients with AIS were treated with Schroth method with or without spinal manipulation at Wenzhou Traditional Chinese Medicine Hospital. Patients were screened based on the inclusion and exclusion criteria. Patients treated with the Schroth method in combination with spinal manipulation were classified as study group, and patients treated with the Schroth method alone were classified as control group. Patients were then matched for gender and finally 50 patients were included in each group.

### Ethical Approval:

This study was conducted with the approval of the Medical Ethics Committee of Wenzhou Traditional Chinese Medicine Hospital (WZY2024-KT-003-01, Date: Feb. 16^th^, 2024). Due to the nature of observation and review, the informed consent was waived.

### Inclusion criteria:

- Patients were examined for AIS through full length spine radiographs.^10^
- Age ranges from 10 to 13 years old.
- Cobb angle between 10° and 20°.
- The clinical data was complete.

### Exclusion criteria:

- Patients with chromosomal abnormalities.
- Patients with peripheral neuropathy and muscle lesions.
- Patients with butterfly vertebrae, hemivertebrae, and syringomyelia.
- Patients with combined growth and development disorders.
- Patients with obvious deformities in their feet and lower limbs.
- Patients with non-idiopathic scoliosis.

### Procedure

### Control group:

**Fig.1:**
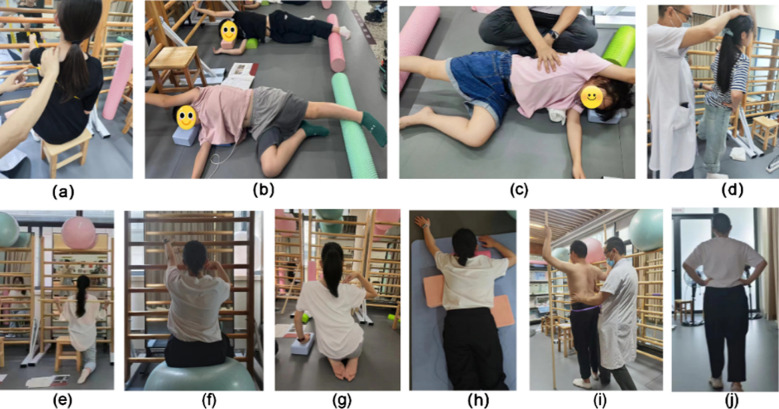
Schroth exercises: (a) rotational respiration and elongation study, (b) shoulder counter-traction lying on the side, (c) muscle cylinder exercises lying on the side, (d) sail exercise, (e) muscle cylinder exercises in the half-kneeling position, (f) sitting on a ball, (g) sideways hangs, (h) shoulder counter-traction in the prone position, (i) prominent hip exercise, (j) Schroth gait exercises.

The Schroth method was performed. In the first training session, patients received training on the basic principles of Schroth, including 3D Schroth rotational breathing exercises, pelvic correction exercises, stretching, basic tension, and positioning. In the same lesson, the patient received shoulder reverse traction, muscle column, side hanging, sail, hip protraction, and Schroth walking exercises (Fig.1). The above intervention lasts for 90 minutes per session, three times a week, lasting for six months.

### Study group:

In addition to the Schroth therapy, spinal manipulation was also performed. The procedures for spinal manipulation were as follows.

### Massage of pressure points:

The Foot Sun Bladder Meridian and Governor Vessel were located. Massage and point pressing were performed along the bilateral bladder meridians of the Du meridian from top to bottom and along the Du meridian from the Dazhui acupoint to the tail. The focus was on massaging the acupoints of Wei Zhong, Qi Hai, Ming Men, Yin Men, and Huan Tiao for five minutes in total, 40 times/min.

### Spasm relief:

Patients were guided to stand, and the most spasmodic and tense areas of the muscles and ligaments at the lateral curvature of the spine were identified. Patients were then guided into the prone position, and muscle pulling on both sides of the spine was performed. The palm backup rolling method, elbow massage, and vibrating techniques were used to relieve muscle and ligament adhesion and spasms. The massage therapy was performed for 10 minutes/time.

### Whole bone manipulation:

Muscles and spinous processes at the lateral bend were sequentially pushed and pulled. Care was taken to ensure gentle and continuous movements, and intensity that the patient tolerates. Manipulation was carried out five minutes/time. After the treatment, the patient was helped into the prone position and instructed to relax the whole body. Standing on the side of the bend, the therapist held down the life gate at the waist with one hand and pulled the shoulder with the other hand. Force was applied with both hands simultaneously, twisting and extending the spine to the maximum extent possible, one minute per session, a total of two times. The waist of the patient was fixed, and the patient’s shoulders were gently shaken using both hands two minutes per time. The treatment was performed twice a week for a period of six months.

### Baseline data and the following relevant indicators were collected before and six months after the treatment:

### Treatment effect:

The treatment effect was determined based on the Cobb angle, measured by X-ray examination of the full length of the spine. Compared to before treatment, the treatment effect was classified as 1) Significant effective: the spine Cobb angle was ≤ 10° or there was a reduction of ≥ 5°; 2) Effective: Cobb angle reduction of ≤ 5°; 3) Invalid: the X-ray examination of the full length of the spine showed an increase or no change in the Cobb angle.^11^ Significant and effective rates were included in the overall effective rate.

### Maximum Cobb angle and clavicle angle:

12 A full-length orthophoto film of the spine was taken in a standing position. The maximum Cobb angle was the angle between the vertical line of the lower vertebral endplate and the vertical line of the upper vertebral endplate. The angle formed by the line connecting the horizontal plane and the highest points of the bilateral clavicles was the clavicle angle.

### Angle of trunk rotation (ATR) and vertebral rotation angle:

The vertebral rotation angle was measured using the Nash-Moe method.^13^ ATR was measured using the Scoliometer.

### Lumbar range of motion (ROM):

The range of motion for lumbar extension and flexion was measured using a protractor.

### Statistical Analysis:

Statistical analysis was conducted using SPSS 20.0 (IBM Corp, Armonk, NY, USA) and PRISM 8.0 software (GraphPad, San Diego, USA). The Shapiro-Wilk test was used to evaluate the normality of data. The data with normal distribution were presented as mean ± standard deviation and analyzed using the *t*-test. Non-normally distributed data were presented as median and interquartile intervals and analyzed using Mann-Whitney U-test. Categorical data were presented as numbers and percentages, and analyzed using the Chi-square test. A *p*-value less than 0.05 was considered statistically significant. All reported p-values were bilateral.

## RESULTS

There was no statistically significant difference in the baseline data such as gender, age, weight, height, lesion location, and Riser sign between the two groups of patients (*P*>0.05) (Table-I). The overall efficacy of the treatment in the study group was significantly higher than that in the control group (96.00% *versus* 84.00%) (*P*<0.05) (Table-II).

**Table-I:** Comparison of baseline data between two groups.

Baseline	Study group (n =50)	Control group (n =50)	t/x²	P
Male /Female	21/29	27/23	1.442	0.230
Age (year)	12(12-13)	11(12-13)	-1.255	0.210
Weight (kg)	37.92±10.77	35.86±11.69	0.916	0.362
Height (cm)	147.74±9.62	146.92±13.08	0.357	0.722
***Location of lesion [n (%)]***				
Thoracic segment	17(34.00)	14(28.00)		
Thoracolumbar	24(45.00)	21(42.00)	1.990	0.370
Lower lumbar segment	9(18.00)	15(30.00)		
***Risser sign [n (%)]***				
0°	4(8.00)	6(12.00)		
I °	13(26.00)	13(26.00)		
II °	18(36.00)	15(30.00)	1.347	0.718
III °	15(30.00)	16(32.00)		

**Table-II:** Comparison of intervention effects between two groups.

Group	n	Significant effect	Effective	Invalid	Total effective rate
Study group	50	29 (58.00)	19 (38.00)	2 (4.00)	48 (96.00)
Control group	50	22 (44.00)	20 (40.00)	8 (16.00)	42 (84.00)
*χ^2^*					4.000
*P*					0.046

**Fig.2:**
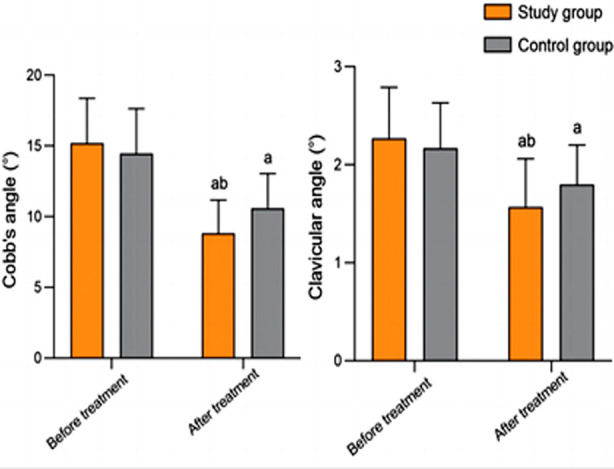
Comparison of maximum Cobb angle and clavicle angle between two groups; Compared to before treatment in the same group, ^a^P<0.05; compared with the control group, ^b^P<0.05.

**Fig.3:**
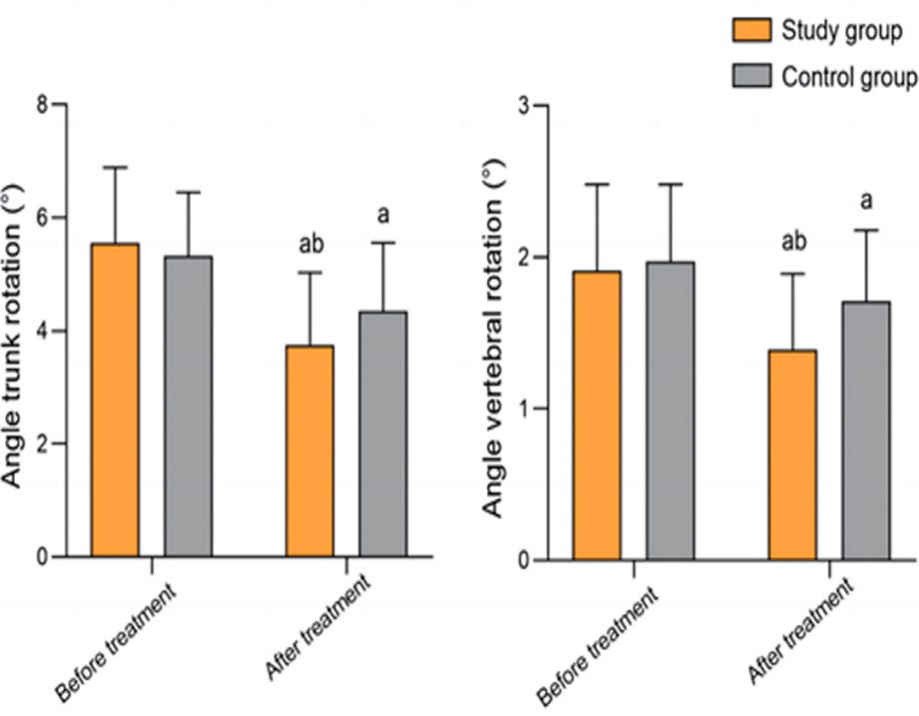
Comparison of trunk rotation angle and vertebral rotation angle between two groups; Compared to before treatment in the same group, ^a^P<0.05; compared with the control group, ^b^P<0.05.

Before the treatment, there was no significant difference in the maximum Cobb angle and clavicle angle between the two groups (*P*>0.05). After the treatment, both angles in the two groups significantly decreased and were significantly lower in the study group compared to the control group (*P*<0.05) (Fig.2). Pre-treatment trunk rotation and vertebral rotation angles were comparable in the two groups (*P*>0.05). After the treatment, both angles markedly decreased in the two groups compared to before the treatment and were significantly smaller in the study group than in the control group (P<0.05) (Fig.3).

**Fig.4:**
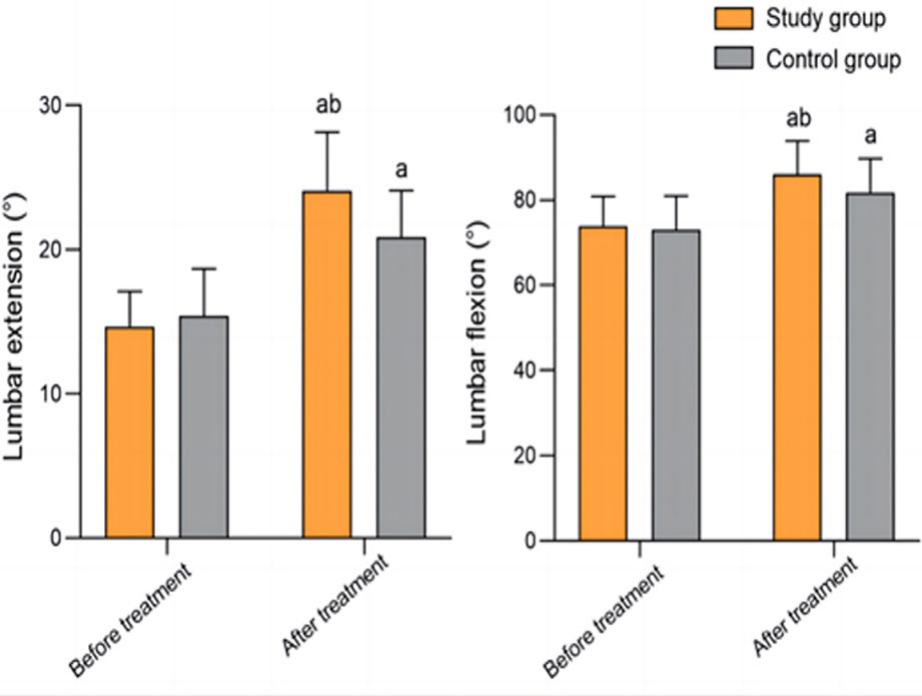
Comparison of lumbar range of motion between two groups; Compared to before treatment in the same group, ^a^P<0.05; compared with the control group, ^b^P<0.05.

Before the treatment, both groups had similar degrees of lumbar extension and flexion (*P*>0.05). While the post-treatment degree of lumbar extension and flexion in both groups significantly increased compared to pre-treatment levels, they were significantly higher in the study group than in the control group (*P*<0.05) (Fig.4).

## DISCUSSION

This study showed that the combination of the Schroth method and spinal manipulation techniques was significantly more effective in treating AIS than the Schroth method alone. The spinal manipulation techniques combined with the Schroth method in this study led to a significantly higher overall efficacy of patients (96.00%) compared to the Schroth method alone (84.00%). Moreover, the combined regimen was associated with significantly lower post-treatment maximum Cobb, clavicle, trunk rotation, and vertebral rotation angles, while the lumbar extension and flexion degrees were greater than those in the control group of patients who were treated by the Schroth method alone. The conclusions are similar with previous studies.^11^,^14^

Numerous clinical studies have confirmed the clinical value of the Schroth method in the treatment of AIS.^15^-^17^ The Schroth method has been shown to be associated with the reduced trunk rotation angle and Cobb angle, which has a certain positive significance in improving the quality of life of patients with AIS.^17^-^19^ Schroth exercise regimen led to a significant improvement in cervical alignment and shoulder balance. Kuru et al.^20^ also confirmed that the Cobb angle and rotation angle of patients with AIS significantly decreased. This study showed that the post-treatment Cobb angle of patients treated with the Schroth exercise regimen was considerably lower compared to pre-treatment levels, which is consistent with previous reports.^17^-^20^

Spinal manipulation is also an important rehabilitation technique in orthopedics and is effective in reducing pain and restoring body function in patients with various musculoskeletal diseases.^8^,^9^ Based on the progression of the disease, local anatomy, and characteristics of muscle imbalance, techniques such as kneading, pressing, tapping, and pushing are used to guide the affected soft tissue, focusing on reconstructing the neck, chest, waist, and sacrum, reducing soft tissue stress, and restoring the dynamic balance of the spinal dynamic system.^21^,^22^

Bilaosky et al.^23^ confirmed the value of spinal manipulation as an alternative medical treatment method and hypothesized that abnormal positions or movements of bones, muscles, and joints may cause physical pain and dysfunction. Therefore, adjusting the position of bones and joints using manual techniques, such as massage and bone setting, may improve mechanical balance, relieve pain, and improve motor function.^23^,^24^ Groisman et al.^25^ also showed that the combination of spinal manipulations for non-specific chronic neck pain could not only alleviate the pain level of patients but also reduce the risk of disability and improve neck mobility. Similarly, Bagagiolo et al.^26^ found that manual bone setting has a positive effect on the treatment of musculoskeletal diseases and was beneficial for alleviating pain and reducing the impact of the disease on the physical and mental health of patients. Stępnik et al.^27^ used spinal manipulations in patients with respiratory diseases and showed that they can regulate thoracic joint mobility, increase peak respiratory flow, and improve respiratory function and quality of life of patients. Our results further confirmed that the combination of the Schroth method and spinal manipulation has efficacy in AIS, helps promote the functional recovery of patients, and positively improves their quality of life.

### Limitations:

This is a single-center small-scale study, which may limit the generalizability of our results. The retrospective study design, small sample size, and lack of power calculation limited the strength of our conclusions. In this study, we only included patients aged 10-13 years old. Although this age group is at high risk for AIS and is most suitable for spinal manipulation treatment, other age groups need to be included in the future to verify the observations of this study. The progression of AIS generally lasts for several years and requires long-term, individualized treatment interventions that may be extended beyond the six months treatment period. However, this study did not carry out the long-term follow-up management of patients. Further studies are needed to determine the impact of the Schroth method combined with spinal manipulations on the long-term functional rehabilitation effect of patients with AIS.

## CONCLUSION

Compared with the Schroth method alone, the Schroth method combined with spinal manipulation intervention can more effectively reduce the maximum Cobb angle, clavicle angle, trunk rotation angle, and vertebral rotation angle and restore lumbar mobility in patients with AIS. A combined treatment regimen, therefore, is associated with improved overall treatment effectiveness.

### Authors’ Contributions:

**HC:** Study design, literature search, manuscript writing, manuscript revision and validation and is responsible for the integrity of the study.

**HC** and **CZ:** Data collection, data analysis, interpretation and critical review.

All authors have read and approved the final manuscript.
